# DNA methylation, a hand behind neurodegenerative diseases

**DOI:** 10.3389/fnagi.2013.00085

**Published:** 2013-12-05

**Authors:** Haoyang Lu, Xinzhou Liu, Yulin Deng, Hong Qing

**Affiliations:** School of Life Science, Beijing Institute of TechnologyBeijing, China

**Keywords:** DNA methylation, Alzheimer's disease, Parkinson's disease, Huntington's disease, amyotrophic lateral sclerosis

## Abstract

Epigenetic alterations represent a sort of functional modifications related to the genome that are not responsible for changes in the nucleotide sequence. DNA methylation is one of such epigenetic modifications that have been studied intensively for the past several decades. The transfer of a methyl group to the 5 position of a cytosine is the key feature of DNA methylation. A simple change as such can be caused by a variety of factors, which can be the cause of many serious diseases including several neurodegenerative diseases. In this review, we have reviewed and summarized recent progress regarding DNA methylation in four major neurodegenerative diseases: Alzheimer's disease (AD), Parkinson's disease (PD), Huntington's disease (HD), and amyotrophic lateral sclerosis (ALS). The studies of these four major neurodegenerative diseases conclude the strong suggestion of the important role DNA methylation plays in these diseases. However, each of these diseases has not yet been understood completely as details in some areas remain unclear, and will be investigated in future studies. We hope this review can provide new insights into the understanding of neurodegenerative diseases from the epigenetic perspective.

## Introduction

Epigenetic refers to the study of mitotically or meiotically heritable changes in gene functions that cannot be explained by changes in DNA sequence. In most cases, it acts as the heritable regulation of DNA transcription by DNA methylation, histone modification and expression of noncoding RNAs. Since R. D. Hotchkiss (Hotchkiss, 1948) discovered that DNA can be methylated at the 5-position of cytosine in 1948, the mysterious veil on epigenetics has been gradually lifted. A study of in discordant twins pointed out that epigenetics can make a difference, even in a pair of monozygotic twins (Fraga et al., 2005). Fraga et al. uncovered that even if sharing a common genotype, twins still showed different penetrance of various diseases such as neurological disorders. Among epigenetic mechanisms, DNA methylation is a crucial epigenetic marker that has been most widely studied. Alterations of DNA methylation are involved in many human diseases including cancer and neurological disorders.

Neurodegenerative diseases are one kind of neurological diseases featuring the progressive loss and even final death of neurons. The specific causes and pathological mechanisms of neurodegenerative diseases have not been completely understood. Recently, a large amount of evidence have emerged to show a shared non-Mendelian property between DNA methylation and neurodegenerative diseases, a connection that has peaked research interest for over two decades.

The main purpose of this review is to provide an overview on the involvement of DNA methylation in the pathology of neurodegenerative disease. We will begin by outlining the concept of DNA methylation, then focus intensively on recent progress made in the study of DNA methylation in four major neurodegenerative diseases: Alzheimer's disease (AD), Parkinson's disease (PD), Huntington's disease (HD), and amyotrophic lateral sclerosis (ALS). We will discuss in depth the relationship between DNA methylation and neurodegenerative diseases, as well as the causal and consequential effect of DNA methylation in these diseases.

## The principle of DNA methylation

As the most widely characterized epigenetic modification, DNA methylation in eukaryotes is found nearly exclusively at cytosine residues. A methyl group is added at the 5-position of cytosine before a guanine and gene silencing is frequently associated with this modification. This dinucleotide unit is always written as CpG, representing a combination of a cytosine, the following guanine and a phosphate group between them. Regions with high concentration of CpGs are called CpG islands, which usually locate in promoter region of genes, a place where most CpGs in the human genome exist. Cytosines are usually not methylated in CpG islands, but for some particular functions, such as X chromosome inactivation, methylation of CpG islands is also required. On the other hand, predominantly located in repetitive or centromeric sequences, CpGs outside CpG islands are usually methylated (Reik et al., 2001; Bird, 2002). Data showed that unmethylated regions of the genome are protected from DNA methylation by a combination of factors involving very high CpG densities and histone modifications, while the remaining bulk of the genome is methylated as the default state (Edwards et al., 2010). CpG methylation within promoter and intragenic sites have been extensively studied and recent interest have also arisen regarding non-CpG methylation, which refers to the methylation that occurs at cytosines of non-CpG dinucleotides, such as CA, CT, or CC. While CpG methylation can occur whenever gene silencing is needed during the life span of a cell, non-CpG methylation is dominantly present in embryonic stem cells (Haines et al., 2001; Dodge et al., 2002; Lister et al., 2009) as well as in neural development (Lister et al., 2013).

**Figure 1:**
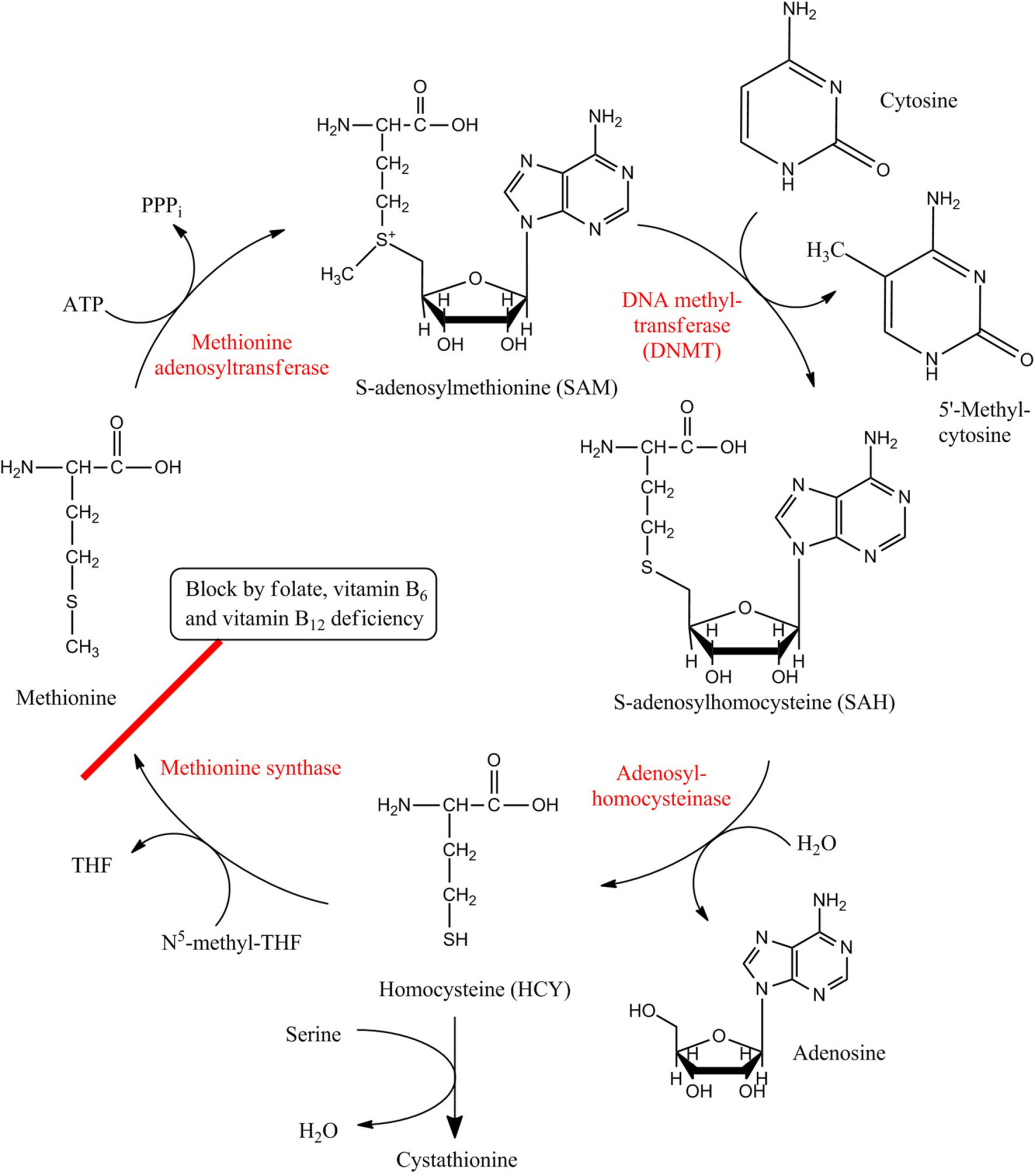
**Metabolic pathway of DNA methylation.** HCY/SAM cycle ensures the continuous transport of methyl group from SAM to cytosine, providing the raw material of DNA methylation. Enzymes are shown in red while substrates and products are shown in black. Red line marks the step where the pathway can be blocked by deficiency of folate, vitamin B_6_ and vitamin B_12_, and such block results in HCY accumulation and other biological effects.

More specifically, 5-methylcytosine (5mC) is produced by transferring a methyl group from an S-adenosyl-L-methionine (SAM) to the cytosine with the help of DNA methyltransferases (DNMTs) (Figure 1). Five kinds of proteins—DNMT1, DNMT2, DNMT3a, DNMT3b, and DNMT3L are major members of DNMT family (Bestor, 2000). The functions of DNMT in DNA methylation can be divided into maintenance methylation and de novo methylation. DNMT1 is involved in maintenance methylation, which refers to the process of copying DNA methylation profiles to the daughter strands during DNA replication. DNMT3a and DNMT3b are de novo methyltransferases that establish DNA methylation patterns in early development. DNMT3L has no catalytic activity but can assist the de novo methyltransferases by improving their ability of binding to DNA and stimulating their activity. Instead of methylating DNA, DNMT2 was shown to methylate the anticodon loop of aspartic acide transfer RNA at cytosine-38 (Goll et al., 2006). Recognizing and binding of methyltransferases to 5-methylcytosines requires methyl-CpG binding domain (MBD) proteins, which are MeCP2, MBD1, MBD2, MBD3, and MBD4 in mammals (Fatemi and Wade, 2006). In addition to 5mC, there exists another kind of methylated cytosine, 5-hydroxymethylcytosine (5hmC), which is consequence of the oxidation of 5mC that is catalyzed by the ten-eleven translocation (TET) proteins. In turn, 5hmC also can be deaminated to 5mC via the mediation of AID/APOBEC family proteins. 5hmC in mammalian DNA was first described in the early 1970s (Penn et al., 1972), however, it has been poorly studied until recently when studies found that 5hmC is present in mouse neurons as well as in embryonic stem cells (Kriaucionis and Heintz, 2009; Tahiliani et al., 2009). Since then, it has been substantiated that 5hmC can influence the regulation of gene expression, and the conversion of 5mC to 5hmC may contribute to DNA demethylation that, in most cases, associates with gene activation (Bhutani et al., 2011; Guo et al., 2011a).

DNA methylation works in harmony with histone acetylation to control memory formation and synaptic plasticity (Miller et al., 2008), and it also has a possible impact on genetic and neuronal function affecting behaviors (Day and Sweatt, 2010). Besides, the connection among DNA methylation, chromatin structure and gene silencing has been extensively studied for many years, and the gene silencing is thought to be an epigenetic intervention on neurodegenerative diseases like AD (Scarpa et al., 2006). Therefore, we can believe that there is a very strong potential link between DNA methylation and neurodegenerative diseases as we will talk about below.

## DNA methylation and alzheimer's disease

### Introduction to alzheimer's disease

AD is the most common form of dementia, which brings acute suffering to its patients. Eleven percent of people age 65 or older and 32% of people age 85 or older are afflicted by this disease. The general symptom pattern begins with the gradually worsening of ability to remember new information. The patient's cognitive and functional abilities decline as the disease progresses. Extracellular neuritic plaques, intracellular neurofibrillary tangles and neuronal loss are the main pathological hallmarks in AD brains. AD is ultimately fatal and has become the sixth leading cause of death in the United States (Thies et al., 2013).

The cause of AD is still unclear. Numerous genetic and environmental risk factors are involved in the etiology and pathogenesis of AD, including alterations in the expression of thousands of genes, amyloid β-peptide (Aβ) deposition, tau hyperphosphorylation, inflammation, oxidative stress, energy metabolism, and aberrant re-entry into the cell cycle/apoptosis. It's worth noting that when Aβ-inducing mutations are absent, these molecular and genetic factors do not have absolute penetrance in causing the disorder (Mastroeni et al., 2011).

Two dominant hypotheses to explain the disease are Aβ hypothesis and tau hypothesis. Redundant Aβ is considered a main contributor to the dysfunction and degeneration of neurons that occurs in AD. Aβ, a 38–43 amino acid peptide, is gained from sequential β- and γ-secretase cleavages of amyloid-β precursor protein (APP). When the cleavage site lies within the Aβ sequence, another APP processing enzyme, α-secretase, precludes Aβ formation. Beta-site APP cleaving enzyme 1 (BACE1) is the major β-secretase in the brain. γ-Secretase consists a minimum 4 proteins: presenilin 1 (PS1) or presenilin 2 (PS2), nicastrin (Nct), presenilin enhancer 2 (Pen2), and anterior pharynx defective 1 (Aph-1) (Chow et al., 2010). Another hypothesis, the tau hypothesis, is based on the hyperphosphorylation of tau in patients with AD. How phosphorylation influences tau function is only poorly understood, but it negatively regulates the binding of tau to microtubules. Hence, functions of tau such as microtubule stabilization and the regulation of axonal transport may be compromised, possibly contributing to disease. Moreover, interactive enhancements between Aβ and tau were also proposed recently (Ittner and Gotz, 2011).

AD can be generally categorized into two divisions. Less than 2% of AD cases are early-onset Alzheimer's disease (EOAD), which onsets prior to age 60 with genetic mutations in APP, presenilin 1, or presenilin 2 genes. Mutations in these genes dysregulate the APP pathway and directly lead to Aβ plaque accumulation, a major pathological hallmark of AD. Other cases which are sporadic and manifest symptoms after age 60, are termed late-onset Alzheimer's disease (LOAD). At least one apolipoprotein 4 allele (APOE-4) is found in approximately 50–65% of LOAD cases, while the global population prevalence of the allele is only approximately 20–25% (van der Flier et al, 2011).

### DNA methylation modification evidence in AD

DNA methylation modifications related to AD can be divided into two groups: global DNA methylation modifications and gene-specific DNA methylation modifications. Global DNA hypomethylation and specific gene hypermethylation are the overall phenomenon among studied AD cases.

#### Global methylation modification and AD

Most studies support the view that on the whole-genome scale, the DNA methylation level in AD cases is lower than in comparison with normal individuals. Direct evidence came from studies in monozygotic twins. A postmortem study of rare sets of monozygotic twins discordant for AD reported significantly reduced levels of DNA methylation in temporal neocortex neuronal nuclei of the AD twin, which provided the potential for AD discordancy in spite of genetic similarities. With specific markers, decrement in DNA methylation was observed in AD reflected among neurons, reactive astrocytes, and microglia (Mastroeni et al., 2009). Research also recognized a dramatic decrease in AD patients of far-going epigenetic markers and regulators in neurons from control entorhinal cortex layer II, consistent with the high vulnerability of this brain region to AD pathology. The result indicates that global DNA and RNA methylation status are significantly diminished in AD in this region (Mastroeni et al., 2010). This trend of global hypomethylation was further confirmed in a more recent and direct study, in which decrements of 5mC and 5hmC were shown in AD patients' hippocampus by a quantitative immunohistochemical method (Chouliaras et al., 2013). A similar result was also shown in a cell line. Using the AD model cell line H4-sw, which harbors the Swedish mutation and produces high levels of the toxic form Aβ, global methylation status were analyzed. Among the total 6296 differentially methylated CpG sites, 23% were shown to be hypermethylated while others were hypomethylated (Sung et al., 2011).

However, such a conclusion is not supported by all the evidence. For instance, a postmortem human frontal cortex genome-wide DNA methylation study also showed general and mild discordant DNA methylation in LOAD-diseased tissue independent of the age factor, while both hypomethylation and hypermethylation were found (Bakulski et al., 2012). Moreover, another postmortem human frontal cortex study showed a trend of global DNA hypermethylation in AD cases (Rao et al., 2012). It is noteworthy that DNA hypomethylation tendency in AD is mainly supported by immunoassays, but the opposite tendency has been observed through bisulfite treatment, which is a direct interrogation of DNA.

#### Gene specific DNA methylation modification and AD

Researchers have also traced the methylation modification of a number of specific genes, which are believed to be related to AD. Such studies usually concentrate on the gene promoter region, in which DNA methylation regulates the expression of genes. We will divide these genes into six sections and introduce findings in each section.

***Aβ-generation-related genes***. APP, PS1, and BACE1 play crucial roles in the generation of Aβ. Therefore, the promoter regions of their genes have been studied in many different researches.

Recent results overall suggest that there is no correlation between APP methylation modification and AD. A large-scale postmortem study involving frontal cortex and hippocampus found no difference in APP gene promoter region methylation among all stages of AD patients and healthy controls. The preservation of DNA methylation status in postmortem brain samples was also confirmed (Barrachina and Ferrer, 2009). Another postmortem study focusing on the cortex and cerebel observed no methylated CpG in APP promoter regions in any familial AD patients and their healthy comparisons (Brohede et al., 2010). However, a study in SH-SY5Y cell lines indicated that the promoter of *APP* is capable to be hypomethylated (Guo et al., 2011b).

The situation of *PSEN1* (the gene encoding PS1) seems more apparent, since almost every study in this field reports *PSEN1* promoter hypomethylation and the resulting overexpression of *PSEN1* as factors leading to AD. Firstly, studies in neuroblastoma cell lines (Fuso et al., 2005) and mouse (Fuso et al., 2008, 2011b) showed that *PSEN1* can be overexpressed through DNA hypomethylation. Further investigations in mouse ruled out the possibility that hypomethylation of *PSEN1* promoter is the consequence of amyloid production (Fuso et al., 2012a). Finally, similar hypomethylation was observed in postmortem study (Wang et al., 2008). However, it should be noted that almost all papers reporting PSEN1 hypomethylation in AD came from the same laboratory, and these results need to be validated by independent laboratories.

It has also been reported that *BACE1* expression can be upregulated via demethylation of at least two CpG sites at position +298 and +351 in the 5′-untranslated region in BV-2 microglial cells (Byun et al., 2012).

***Aβ-degradation-related genes***. Aβ peptides are proteolytically degraded within the brain mainly by neprilysin (NEP) (Iwata et al., 2000) and insulin-degrading enzyme (IDE, insulysin) (Kurochkin and Goto, 1994). The endolytic degradation of Aβ peptides within microglia by NEP and related enzymes is significantly enhanced by apolipoprotein E (ApoE). Similarly, Aβ extracellular degradation by IDE is facilitated by ApoE (Jiang et al., 2008). There are three major isoforms of ApoE: ApoE2, ApoE3, and ApoE4. Among them, ApoE3 is the most common isoform in the population, while ApoE4 has been shown to confer dramatically increased risk for LOAD (Roses et al., 1995).

It has been reported that Aβ causes *NEP* promoter region hypermethylation, which consequently suppresses *NEP's* expression in mRNA and protein levels, reduces the Aβ clearance and probably elevates Aβ accumulation. This could be part of a vicious cycle which plays a role in the pathophysiology of AD (Chen et al., 2009). Sortilin-related receptor (SORL1, also known as SORLA, SORLA1, or LR11) is a neuronal ApoE receptor. Its connection with AD is based on its reduction in AD brains and its ability to lower Aβ levels (Offe et al., 2006). The SORL1 gene showed differences in its expression among peripheral blood leukocytes, and it may act as a marker of aging in this tissue. It has been shown that *SORL1* promoter DNA methylation could serve as one of the mechanisms in charge of the differences in expression observed between blood and brain for both healthy elders and AD patients groups (Furuya et al., 2012a).

***Tau-related genes***. Activity and expression level of glycogen synthase kinase 3β (GSK3β), the major kinase that phosphorylates tau in the brain, are increased in AD brains (Nicolia et al., 2010).

A study in human neuroblastoma SK-N-BE cell line and TgCRND8 mouse showed that GSK3β gene's promoters can be hypomethylated by inhibiting methylation activity through B vitamin deficiency and such hypomethylation results in the overexpression of GSK3β (Nicolia et al., 2010). It was also reported that inhibition of GSK3β reduces the expression of DNMT3A and causes hypomethylation of related genes in mouse embryonic stem cells (Popkie et al., 2010).

***Genes involved in metabolic pathways***. Another finding is that rare haplotypes may be associated with the risk of AD through a possible modulation of the methylation of the ornithine transcarbamylase (OTC) promoter. In spite of being mostly speculative, it might suggest a deregulation of urea cycle in AD (Bensemain et al., 2009).

***Ribosomal DNA (rDNA)***. Ribosomal deficits are verified in mild cognitive impairment (MCI), which often represents an early stage AD as well as the potential of advanced AD (Ding et al., 2005). Although an early study didn't detect any differential methylation pattern of rDNA genes in total peripheral blood cells in elderly and AD subjects (Speranca et al., 2008), it was reported later that the rDNA promoter become hypermethylated in studied AD cerebrocortical samples, suggesting that rDNA hypermethylation could be implicated in AD (Pietrzak et al., 2011).

***Other genes***. The complexity of AD and epigenetics leads to an endless stream of research. One study suggested that behavioral and psychological symptoms in dementia patients may be caused by the methylation disturbance of circadian gene (Liu et al., 2008). In addition, DNA methylation status of repetitive elements long interspersed element-1 (*LINE-1*) was shown to be increased in AD patients compared with healthy volunteers (Bollati et al., 2011). Moreover, various potential functions of neuroglobin (NGB) have been exhibited and found to be able to reduce the severity of stroke and AD (Khan et al., 2007). A 5-aza-2′-deoxycytidine (a methyltransferase inhibitor) treatment analysis indicated that DNA methylation/demethylation may be involved in regulating NGB tissue-specific expression (Zhang et al., 2011). Another separate research implies that long telomeres with hypomethylation tend to shorten faster, while cells bearing short telomeres with hypomethylation tend to enter into a senescent state under elevated OS stress in AD more easily. It has been validated that this trend can be reversed by vitamin E (Guan et al., 2012). Rao et al. reported hypomethylated cyclooxygenase-2 (*COX-2*) (one of the arachidonic acid (AA) cascade markers) and brain-derived neurotrophic factor (*BDNF*) promoter regions in AD brain, while the promoter region of cAMP response element-binding protein (*CREB*), which regulates the transcription of *BDNF*, was shown to be hypermethylated. Their study also found a significant increase in DNA methylation at the promoter region of synaptophysin (*SYP*) and decreased methylation of the NF-kB promoter CpG region in the AD (Rao et al., 2012). S100A2 is a member of the S100 family of calcium binding proteins while *SORBS3* encodes a cell adhesion molecule expressed in neurons and glia. The decrement of *S100A2* and increment in *SORBS3* CpG methylation in AD brains were reported from a postmortem study (Siegmund et al., 2007).

Some other genes involved in AD were also investigated, but no DNA methylation differences have been found in these genes. These genes include synaptosomal-associated protein 25 (*SNAP-25*) (Furuya et al., 2012b), *SIRT3*, *SMARCA5*, *CDH1* (Silva et al., 2008), 2′,3′-cyclic-nucleotide 3′-phosphodiesterase (*CNP*), and dihydropyrimidinase-like 2 (*DPYSL2*) (Silva et al., 2013).

## DNA methylation and parkinson's disease

### Introduction to parkinson's disease

PD is the second most common neurodegenerative disorder after AD. According to the PD Foundation, about 1 million people in the United States and more than 4 million people worldwide are affected with this disease. The prevalence of PD in industrialized countries is generally estimated at about 1–2% of population over 60 years of age, and increases to 3–5% in people above 85 years old. This neurodegenerative disorder is characterized by the progressive loss of substantia nigra dopaminergic neurons and striatal projections, causing the typical symptomatology: muscle rigidity, bradykinesia, tremor, and postural instability (de Lau and Breteler, 2006).

Although more than 90% of the cases can be interpreted as sporadic PD, the greatest insights into PD etiology have come from the study of familial forms. In recent years, mutations in six genes have been identified as causes of PD: *SNCA* (which encodes α-synuclein), *PARK2* (parkin), *PINK1* (PTEN-induced kinase protein 1), *UCHL1* (ubiquitin carboxyl-terminal hydrolase isozyme L1), *DJ1* (protein DJ-1), and *LRRK2* (leucine-rich repeat serine/threonine-protein kinase 2). The hallmark neuropathological sign of PD is the presence of fibrillar aggregates of misfolded α-synuclein called Lewy bodies, which accumulate in the same sites where neuronal loss is found (Urdinguio et al., 2009).

Although PD is less studied than AD in terms of the relation to epigenetics, this section aimed to review the interrelation of PD and DNA methylation is organized into two parts. First part is exploring what genetic factors of PD pathogenesis might be affected by DNA methylation, and the other one is aimed to showing how environmental factors could get involved in the alteration of DNA methylation leading to PD.

### Genetic factors, DNA methylation and PD

#### DNA methylation regulation of α-synuclein

Considering that α-synuclein makes a major contribution to the formation of Lewy bodies and even to the entire pathogenesis of PD, we start from discussion on impact DNA methylation has on α-synuclein.

An earlier study revealed that the DNA methylation pattern within the α-synuclein (*SNCA*) gene promoter region was altered in the blood samples of patients with alcoholism, which was significantly associated with their increased homocysteine levels (Bonsch et al., 2005). This is the first study, to the best of our knowledge, to show a correlation between DNA methylation and α-synuclein in certain syndromes. Later on, it has been reported that the methylation of human *SNCA* intron 1 decreased gene expression while inhibition of DNA methylation activated its expression in the brains of PD patient (Jowaed et al., 2010), which further strengthens the link. Although analysis of postmortem brain did not reveal regional specific methylation differences in the putamen and anterior cingulate between PD and healthy individuals, methylation was found specifically and significantly reduced in the substantia nigra of PD patients (Matsumoto et al., 2010). In addition, single CpG analysis reflected fluctuating methylation levels at different locations in various brain regions and LBD stages, even if the overall methylation levels in the promoter and intron 1 of α-synuclein gene were reported to be similarly low both in Lewy body disease (LBD) patients and controls(de Boni et al., 2011). These results suggest a potential role of DNA methylation in α-synuclein neuropathogenesis.

Unfortunately, in the blood cell sample of a PD patient with heterozygous *SNCA* A53T mutation, α-synuclein expression was found to be monoallelic because of epigenetic silencing of the mutated allele through histone modification instead of DNA methylation (Voutsinas et al., 2010). Rather than altering DNA methylation, α-synuclein negatively regulated PKC-delta expression in human dopaminergic neurons by reducing histone modification (Jin et al., 2011). Moreover, no differential methylation of *SNCA* was observed in white blood cell DNA of PD patients compared to the neurologically normal controls (Richter et al., 2012).

Nonetheless, the interaction of DNMT and α-synuclein has also been tested. Reduction of nuclear DNMT1 levels was observed in postmortem brain samples from PD patients and in the brains of α-synuclein transgenic mice, underlying a mechanism in which DNMT1 might be excluded from the nucleus by α-synuclein, and the segregation of DNMT1 further resulted in hypomethylated CpG islands upstream of α-synuclein(Desplats et al., 2011).

Overall, these results indicate that there is a good chance that DNA methylation is involved in the regulation of α-synuclein gene expression, even if its specific role still remains to be further clarified.

#### DNA methylation in other genes related to PD

In addition to α-synuclein, several other genes also have been examined to find whether they are associated with PD in a DNA-methylation-regulated way.

Tumor necrosis factor α (TNF-α) is a critical inflammatory cytokine and increased TNF-α is associated with dopaminergic cell death in PD. It has been suggested that a lesser extent of methylation of the *TNF-α* promoter in human substantia nigra cells could uphold the increased vulnerability of dopaminergic neurons to TNF-α regulated inflammatory reactions (Pieper et al., 2008). Because TNF-α overexpression induces apoptosis in neuronal cells and TNF-α levels were rather high in the cerebrospinal fluid of PD patients (Mogi et al., 1996), we can speculate that DNA methylation might be the reason for such overexpression of TNF-α. As is well known, the parkin gene plays a relatively important part in the emergence and development of PD. The methylation levels of the parkin gene promoters were analyzed in samples from PD patients heterozygous for *parkin* mutations, PD patients without *parkin* mutations, and normal controls, however, no significant difference was observed among the three groups, indicating that *parkin* promoter methylation alone is unlikely to impact the pathogenesis and development of PD (Cai et al., 2011). Ubiquitin c-terminal hydrolase L1 is a member of one subfamily of deubiquinating enzymes that remove ubiquitin from ubiquitinated substrates in the ubiquitin proteasome degradation system. Dysfunction of UCHL-1 has been implicated in the pathogenesis of neurodegenerative disorders, including PD. The *UCHL1* promoter was found hypermethylated in diverse types of cancer (Kagara et al., 2008; Yu et al., 2008), however, analysis of *UCHL1* promoter in the hippocampus and frontal cortex of PD patients and controls displayed no significant differences in CpG methylation between these two groups (Barrachina and Ferrer, 2009). A large-scale sequencing analysis of postmortem brain samples identified methylation and expression changes associated with PD risk variants in *PARK16/1q32*, *GPNMB/7p15*, and *STX1B/16p11* loci, suggesting that some other PD-related genes could also be epigenetically modified in PD brains (Plagnol et al., 2011).

More recently, it was reported that the expression of clock genes was altered in both PD patients and in animal models of the disease (Cai et al., 2010; Hood et al., 2010). Promoter methylation analysis of seven clock genes in blood samples of PD revealed that most clock gene promoters were short of methylation, suggesting that altered promoter methylation may contribute to the aberrant expression of clock genes in PD (Lin et al., 2012). A genome-wide methylation analysis of PD with quantities DNA methylation levels found reduced methylation of the cytochrome P450 2E1 gene in PD patients' brains compared to the controls, which suggests that epigenetic variants inde-toxifying enzymes, such as cytochrome genes, may add to PD susceptibility (Kaut et al., 2012). Mesodiencephalic dopaminergic (mdDA) neurons, located in the ventral mesodiencephalon, are involved in severely affected neurodegenerative diseases such as PD. Emerging evidence shows that epigenetic mechanisms including DNA methylation play an important role in mdDA development, maturation and maintenance (van Heesbeen et al., 2013). Furthermore, other specific genomic regions were also examined on the methylation level. For example, researchers have found that the number of short telomeres with constant subtelomeric methylation status was smaller in peripheral leukocytes from Japanese PD patients compared to the healthy controls (Maeda et al., 2009). Although, based on the limited number of studies in this field, we cannot come up with a meaningful conclusion from these findings, but at least there might be a bold hypothesis that DNA methylation associates with PD through a variety of genetic pathways.

### Environmental factors and DNA methylation in PD

Environment can significantly affect the risk and progression of neurodegenerative disorders including PD. Here we synthesized published studies focused on the interaction between DNA methylation and environmental factors in PD cases.

Elevated plasma homocysteine levels have been documented in PD individuals (O'Suilleabhain et al., 2004). A further increase in plasma homocysteine levels of blood cell samples was observed in individuals with both PD and the *MTHFR* C677T mutation (Brattstrom, 2001). PD patients undergoing regular treatment with L-Dopa had higher plasma homocysteine concentrations relative to healthy controls, indicating a possible methylated catabolism of the drug (Blandini et al., 2001). Dietary folate deficiency and elevated homocysteine levels have been found to be harmful to dopaminergic neurons in mouse models of PD (Duan et al., 2002). A recent study found out that hallmarks of neurodegeneration such as APP and α-synuclein were related to markers of methylation like SAM and its downstream byproduct, S-Adenosyl-L-homocysteine (SAH) in individuals with PD (Obeid et al., 2009). A higher SAM/SAH ratio, which indicates a higher methylation potential, was linked to better cognitive function. Note that the SAM/SAH ratio is a significant positive predictor of DemTect scores in PD patients and DemTect is a cognitive screening instrument sensitive to the early cognitive symptoms of dementia including AD and PD (Kalbe et al., 2004).

Telomeric dysfunction has been discovered to be associated with development of age-related pathologies, and similarly to AD, shortened telomeres were found present in patients with PD (Guan et al., 2008). Telomere length is epigenetically regulated by DNA methylation, which in turn could be modulated by folate status. In human, telomere length has been reported to be associated with folate status (Paul et al., 2009). Plus, various nutrients also showed potential to influence regulation of telomere length, e.g., folate, via its role in epigenetic status of DNA methylation and histone modification (Paul, 2011).

Besides, environmental exposure, including paraquat, is believed to be a risk for PD. Interestingly, a pretreatment of PC12 with 5-aza-2′deoxycytidine, a DNMT inhibitor, sensitizes cells to paraquat exposition. Similar results were obtained using dopaminergic cells and treatments of MPP(+), 6-hydroxydopamine and rotenone, (Wang et al., 2013) suggesting that DNA methylation might modulate the effect of these toxins and might play a role in PD susceptibility (Kong et al., 2012). These findings underlie a possible mechanism in which environment influences pathology of PD via DNA methylation modification. Nevertheless, since data presented so far are too insufficient to substantiate any hypothesis, further solid findings are very necessary and helpful to arrive at a reliable conclusion.

## DNA methylation and huntington's disease

### Introduction to huntington's disease

HD, or Huntington's chorea, is the most common genetic cause of chorea in high-income countries, with a prevalence of about one in 10,000 people. This lethal neurodegenerative disease primarily affects the cerebral cortex and the striatum. Initial physical symptoms are chorea, rigidity, and dystonia, and become more apparent as the disorder progresses. Cognitive abilities become gradually impaired, finally leading to dementia (Babenko et al., 2012).

HD is caused by the expansion of CAG triplet repeats in the *HTT* gene, which encodes an expanded polyglutamine (polyQ) stretch in the huntingtin (HTT) protein. Normal *HTT* genes contain CAG repeats no more than 35, and are not associated with the disorder. Incomplete penetrance happens with 36–40 repeats. However, when these repeats reach 41 or more, the disease becomes completely penetrant. The number CAG repeats accounts for ~60% of the variation in age of onset, and the rest can be explained by modifying genes and environment (Walker, 2007).These expanded polyQ sequences in the HTT protein produce aggregates that form intracellular inclusions, leading to neural loss, particularly in the caudate nucleus (Rubinsztein and Carmichael, 2004).

Most studies aimed to find a clear correlation between HD and DNA methylation focus on two specific subjects: *HTT* gene and triplet repeat expansions. In addition to the genetic factors, environment also plays a vital role in this DNA methylation-associated mechanism.

### Genetic factors, DNA methylation, and HD

Researches on DNA methylation in HD began much earlier than those in PD. In 1988, Wasmuth et al. reported the identification of a highly polymorphic locus, D4S95, which was demonstrated to be tightly linked to the HD gene (Wasmuth et al., 1988). Later, methylation was found at the B5 allele of the D4S95 locus, which was not inherited in a Mendelian fashion, as its appearance depended on the methylation status of the human lymphoblastoid cells from which DNA samples were obtained (Pritchard et al., 1989), and such phenomenon disclosed the secret of the role of DNA methylation in HD. For the first time, Farrer et al., in their study of 1764 HD patients explored that DNA methylation might be involved in a genetic imprinting mechanism, and thus responsible for the expression of HD (Farrer et al., 1992). Although a comparison between HD patients and normal controls showed no strong relevance between methylation and onset age of the disease, a significant association of the patient's age with demethylation at D4S95 was found (Reik et al., 1993). A PCR-amplication of synthetic oligodeoxyribonucleotides revealed that cytidine methylation could have an impact on the expansion of triplet repeat sequences (Behnkrappa and Doerfler, 1994). Another finding that genome-wide demethylation could accelerate instability of CTG/CAG trinucleotide repeats in mammalian cells, implies that changes in methylation patterns during epigenetic reprograming may trigger the intergenerational repeat expansions, leading to neurological diseases like HD (Gorbunova et al., 2004).

It has been discovered that there is a region of highly unstable CAG repeats at the human spinocerebellar ataxia type 7 (SCA7) locus, and this region contains binding sites for *CTCF*, a regulatory factor involved in genomic imprinting, chromatin remodeling, and DNA conformation change (Filippova et al., 2001). Recently, an investigation in transgenic mice model found that CpG methylation of *CTCF* binding sites could further destabilize triplet repeat expansion (Libby et al., 2008), underpinning the role of DNA methylation in the regulation of neurological diseases. The HD-associated modification of BDNF gene expression was found, in the hippocampus of female and male HD mice independent of methylation increases in the gene sequence, while there existed a pattern of sex-specific differences in the levels of methylation at individual CpG sites, suggesting that such differences might explain the differential regulation of *BDNF* expression in the male and female brains (Zajac et al., 2010). Since it has been reported that the loss of BDNF gene transcription is likely a central factor to the progressive pathology of HD, DNA methylation might be the explanation of such loss, which, however, needs further confirmation. Moreover, extensive changes in DNA methylation were reported to be linked to expression of mutant huntingtin gene, revealing the potential effects of DNA methylation alterations on neurogenesis and cognitive decline in patients with HD (Ng et al., 2013).

Besides, a recent study focused on adenosine A_2A_ receptor (A_2A_R), a G-protein-coupled receptor, the expression levels of which are sharply reduced in HD (Villar-Menendez et al., 2013). The study found increased 5mC levels and reduced 5hmC levels in 5′UTR region of A_2A_R gene from HD patients compared to age-matched controls, suggesting an involvement of an altered methylation pattern of *A*_*2A*_*R* gene in HD pathology. Moreover, instead of methylated cytosine, a HPLC-based method also detected levels of 7-methyl guanine in DNA samples both from transgenic mice and HD patients, revealing aberrant methylation levels in HD (Thomas et al., 2013), and also widening the range of future researches on DNA methylation.

## DNA methylation and amyotrophic lateral sclerosis

### Introduction to amyotrophic lateral sclerosis

ALS is an idiopathic, fatal neurodegenerative disease of the human motor system. The clinical hallmark of ALS is the presence of the lower motor neuron signs in brainstem and spinal cord, and the upper motor neuron signs in the motor cortex. Loss of these neurons leads to clinical phenotypes including muscle atrophy, weakness, fasciculation, spasticity, and cognitive dysfunction (Kiernan et al., 2011). Proposed pathogenic mechanisms for ALS include oxidative stress, glutamate excitotoxicity, impaired axonal transport, neurotrophic deprivation, neuroinflammation, apoptosis, and altered protein turnover, etc. Furthermore, influences from astrocytes and microglia in the motor neuron microenvironment contribute to ALS pathogenesis (de Carvalho and Swash, 2011).

ALS is traditionally classified into two categories: familial ALS (FALS) and sporadic ALS (SALS) (Robberecht and Philips, 2013). FALS is predominantly hereditary and then almost always autosomal dominant, while X-linked or recessive FALS is rare. Several genes and their mutations have been found to be associated with ALS. Superoxide dismutase 1 (*SOD1*) mutations are the cause in about 20% of FALS. Also, *TARDBP*, which encodes TAR DNA-binding protein, and *FUS*, a RNA- binding protein fused in sarcoma, also contribute to FALS cases. SALS has been associated with another gene, *ELP3*, encoding the catalytic subunit of the histone acetyltransferase (HAT) complex elongator protein (Urdinguio et al., 2009). Additionally, *ALS2*, *ATXN2*, and some other genes are also associated with ALS, which we will discuss below (Ferraiuolo et al., 2011).

Not unlike those neurodegenerative diseases we described above, there is a potential point that ALS is also connected to DNA methylation through genetic and environmental factors.

### Genetic factors, DNA methylation, and ALS

An epigenetic analysis of *SOD1* and *VEGF* (which encodes vascular endothelial growth factor, a signal protein produced by cells that stimulates vasculogenesis and angiogenesis) in ALS showed that the promoter regions of these genes were widely unmethylated in ALS patients, suggesting transcriptional silencing via DNA methylation is not likely a common mechanism in ALS (Oates and Pamphlett, 2007).

In contrast, methylation of the human glutamate transporter EAAT2 gene promoter has been reported to be associated with the silent state of the human *EAAT2* gene. Since the dysfunction of EAAT2 transporter might contribute to the pathogenesis of ALS (Rothstein et al., 1995), it is meaningful to further test the regulation of EAAT2 transporter via an epigenetic mechanism including DNA methylation in ALS models. GLT1 is the analog to EAAT2 in rodent astroglial cells, however, hypermethylation on specific CpG islands of *GLT1* promoter was discovered to participate in repression of GLT1 promoter activation, whereas this regulation was not involved in astroglial dysfunction of EAAT2 in ALS patients (Yang et al., 2010). Studies also found that a group of genes, either unsuspected in SALS or in potential pathways of cell death, revealed changed methylation status in SALS brains (Morahan et al., 2009). Because of the controversial findings above, there are supposed to be more powerful and convincing evidence collected to clarify the involvement of DNA methylation in ALS by regulating the expression of the human EAAT2.

In mouse models, the apoptosis process of motor neurons showed alterations in DNMT1, DNMT3a, and 5-methylcytosine, which is similar to those in human ALS, indicating that DNMT may mediate neuronal cell death through DNA methylation (Chestnut et al., 2011). In addition, CpG methylation in human ATXN2 gene promoter is associated with pathogenic CAG expansions in spinocerebellar ataxia type 2 (SCA2) cases (Laffita-Mesa et al., 2012). Since such aberrant expansions in *ATXN2* were shown to contribute to ALS (Lahut et al., 2012), we can expect that there might exist a link between *ATXN2* promoter methylation and pathogenesis of ALS.

### Environmental factors and DNA methylation in ALS

Environmental exposure to heavy metals has been implicated in SALS and functionally impaired detoxification of these metals may cause serious susceptibility to the disease. The metallothionein (MT) is a family of proteins that are involved in primary detoxification mechanism for heavy metals. As a matter of fact, no promoter methylation of human MT genes was evident in any SALS or control samples, implying the possibility that methylation at these gene promoters may not be a common cause of SALS (Morahan et al., 2007). However, altered methylation of the Alsin (ALS2) gene promoter was observed in hippocampal cells of individuals with a history of being abused in childhood (Labonte et al., 2012). Interestingly, higher levels of promoter methylation were correlated with a repression of ALS transcription suggesting a role of DNA methylation in the regulation of ALS gene.

## Discussion

We find three topics worth discussing: (1) causal relationship between DNA methylation modification and neurodegenerative diseases, (2) triggers of DNA methylation modification in neurodegenerative diseases, and (3) perspectives.

In the field of neurodegenerative diseases, although experimental evidence revealed correlations between DNA methylation and these diseases, two major questions remain unclear. The first question is the causal relationship between DNA methylation modifications and neurodegenerative diseases. In other words, do DNA methylation modifications precede the appearance of neurodegenerative symptoms? Is there a proved mechanism demonstrating that DNA methylation modifications will finally lead to neurodegenerative diseases? Another question is: if DNA methylation modifications do cause neurodegenerative diseases, what is the trigger of these methylation modifications in neurodegenerative diseases? This is also a crucial question since it may lead us to new paths of curing these diseases.

### Causal relationship between DNA methylation modification and neurodegenerative diseases

#### Alzheimer's disease

Evidences generally suggested that DNA methylation modification is a cause instead of a consequence of AD, for several events related to DNA methylation occur earlier than AD symptoms.

One clue is that the upregulation of plasma homocysteine (HCY), an independent AD risk factor, occurs prior to the pathogenesis of AD (Clarke et al., 1998; Seshadri et al., 2002). As we know, DNA methylation is accomplished by transferring a methyl group from SAM to the 5-position of cytosine. This metabolic pathway belongs to HCY metabolism (one-carbon metabolism). HCY accumulation leads to upregulation of S-adenosylhomocysteine (SAH) levels due to the reversibility of the reaction. To make the reaction proceed in the hydrolytic direction, HCY and Ado have to be efficiently removed (Fuso et al., 2005) (Figure 1). SAH, a strong DNA methyltransferase inhibitor, strengthens DNA hypomethylation. Thus, a regulation of metabolism through either remethylation or transsulfuration pathways may result in hyperhomocysteinemia, decrease of SAM/SAH ratio (also called methylation potential, MP), and change of GSH levels, suggesting that hypomethylation is a mechanism through which HCY is related to vascular disease and AD. Additionally, oxidative stress was shown to promote the production of HCY's oxidized derivatives, such as homocysteic acid and homocysteine sulfinic acid. These compounds interact with glutamate receptors thus increasing intracellular free radicals (Fuso and Scarpa, 2011). Following this trail, a series of studies demonstrated PS1 gene promoter hypomethylation in cell and mouse models under alterative HCY case (Scarpa et al., 2003; Fuso et al., 2005, 2007, 2008). These cases are often accomplished by deficiency of vitamin B6, vitamin B12 and folate during cell culture and mouse feeding. Moreover, in SK-N-BE neuroblastoma cells and TgCRND8 mice, such a trend is found to be reverted when SAM was intentionally added to the culture or diet (Fuso et al., 2011b). Cognitive experiments in mice and detection of Aβ formation confirmed these results (Fuso et al., 2012b). It has also been shown that the activities of DNA methyltransferases (DNMT1, DNMT3A, DNMT3B) and demethylase (MBD2) are modulated by HCY metabolism in AD cells and mouse models. Elevated HCY levels decreased the activity of DNA methylase and the activity of DNA demethylase was increased (Fuso et al., 2011a). Since the elevation in the homocysteine level preceded the onset of dementia, studies from this area implies that global DNA hypomethylation is a cause of AD.

DNA methylation regulation in aging offers another clue to the causal relationship. Aging is generally considered to be one of the most salient risk factors for AD, and a strong correlation between DNA methylation regulation and aging was demonstrated in brain and blood samples (Horvath et al., 2012). Decrement of *S100A2* and increment in *SORBS3* CpG methylation in the human cerebral cortex during aging was reported, and an acceleration of this trend was shown in AD patients (Siegmund et al., 2007). Another study including 24 LOAD brains and 10 matched controls revealed epigenetic variability of genes related to AD among all individuals, and AD patients' epigenetic distance from the norm was observed to increase progressively with age. It was suggested that epigenetic modifications may merely result in a range of interindividual variance until a threshold of epigenetic deregulation is reached. After this point, the brain starts to malfunction and AD symptoms occur. Based on this view, LOAD may represent an extreme form of normal aging (Wang et al., 2008). To sum up, these studies indicated that methylation changes are parts of aging, and some of them may result in AD.

It is noteworthy that there are results from another side. In a murine cerebral endothelial cells model, it was shown that Aβ reduces global DNA methylation whilst increasing *NEP*'s DNA methylation level and further suppressing *NEP*'*s* expression in mRNA and protein levels (Chen et al., 2009). This finding suggested a vicious cycle formed by DNA methylation alteration and Aβ production, in which these two phenomena reinforce each other and cause AD eventually. This point is interesting, however, most other studies of this kind showed the opposite result. For instance, a recent study showed that amyloid production was not responsible for PS1 demethylation in the brain of TgCRND8 mice (carrying a double-mutated human APP transgene) (Fuso et al., 2012a). Therefore, such results need to be further analyzed.

#### Parkinson's disease, huntington's disease, and amyotrophic lateral sclerosis

Similar to AD, the other three major neurodegenerative diseases, i.e., PD, HD, and ALS, are also closely related to DNA methylation of several critical genetic loci. While these loci have been extensively studied, some findings seem to disagree with others. In the case of PD, α-synuclein gene is the research spot of interest. There have been a series of studies of human PD brain cell samples that tested the DNA methylation level of the promoter and intron 1 of α-synuclein gene and found a different methylation level compared to normal controls (Jowaed et al., 2010; Matsumoto et al., 2010; de Boni et al., 2011). These findings all support the idea that DNA methylation is involved in PD. However, some other results are not so positive as they found, while examining blood cell samples of PD patients, that α-synuclein expression was not changed (Richter et al., 2012), or its expression changed independent of DNA methylation modification (Voutsinas et al., 2010). This seems a little controversial, but if we take a closer look at these results against the involvement of DNA methylation in PD, we can find that these examinations taken with blood cell samples are much less convincing than those taken with brain cell samples in that α-synuclein is widely believed to mainly be localized and functional in mammalian brain neurons (Mclean et al., 2000; Yu et al., 2007). Other genetic loci with issues are parkin and UCHL1 genes, which also play important roles in PD pathology, however, due to lack of enough evidence so far, we still cannot determine whether these loci are affected by DNA methylation or not.

In the case of HD and ALS, the studies were merely focused on the rough relationship between these diseases and DNA methylation. Although, of course, there are some genetic loci that also have been examined in those researches such as CTCF, BDNF, and ATXN, we can barely come to any specific conclusion because of the poor quantity of scientific evidence.

### Triggers of DNA methylation modifications in neurodegenerative diseases

#### Alzheimer's disease

Knowing that DNA methylation changes do interact with AD and usually serve as causes, one may seek the origin of these changes in DNA methylation patterns. Such pursuit is worthwhile since it may finally guide us to a new path for treating AD. We divide possible causes of DNA methylation regulation in AD into four aspects: aging, B vitamin deficiency, oxidative stress, and heavy metal exposure.

***Aging factors***. As we mentioned above, aging is a widely-accepted risk factor of AD, and some reports regarded methylation change related to AD as an acceleration of aging (Siegmund et al., 2007) or a specialized case of aging (Wang et al., 2008). These studies provide a chain of causation from aging to DNA methylation deregulation and then to AD.

***B vitamin deficiency***. As we know, DNA methylation is accomplished by transferring a methyl group from SAM to the 5-position of cytosine and this metabolic pathway belongs to HCY metabolism (one-carbon metabolism). Folate, vitamin B_6_ and vitamin B_12_ are crucial for this metabolic cycle since N^5^-methyl-tetrahydrofolate (N^5^-methyl-THF, a folate derivative) donates a methyl group to HCY that then transforms to methionine while vitamin B_6_ and vitamin B_12_ are involved in methylation catalysis. Therefore, the deficiency of vitamin B may block the regular DNA methylation metabolic cycle. This indication is supported by experimental evidence in rats (Miller et al., 1994), and B vitamin deficiency is commonly used as a method to create DNA methylation metabolism disorder in current studies (Fuso et al., 2005, 2011b; Chen et al., 2009). In addition to those hypermethylation and hypomethylation patterns we mentioned above, it was demonstrated that SAH increased DNA damage in BV-2 cells possibly by increasing Aβ formation that led to increased formation of ROS. Furthermore, the DNA damage was reinforced by SAH through inhibition of DNMT1 activity and hypomethylation of OGG1 gene promoter in microglial BV-2 cells (Lin et al., 2011).

Moreover, HCY inhibits the dimerization of ApoE3 and reduces ApoE3-mediated high-density lipoprotein (HDL) generation (Minagawa et al., 2010). It was shown that HDL apolipoproteins can significantly enhance the degradation of soluble Aβ within microglia, and such degradation was facilitated by the lipidation of ApoE (Jiang et al., 2008). HCY was reported to impair ApoE3 dimerization and ApoE3's ability of generating HDL by binding to cysteine residues of ApoE3. Therefore, hyperhomocysteinemia may promote the pathogenesis of AD (Minagawa et al., 2010).

***Oxidative stress***. Causing the imbalance between DNA methylation and demethylation, oxidative stress is also known as an environmental factor interacting with DNA methylation and AD. Study in SH-SY5Y cells revealed that treatment with H_2_O_2_ may activate a DNMT inhibitor and result in the upregulation of APP and BACE1 through transcription activator-vB, leading to the upregulation of Aβ production (Gu et al., 2013).

***Heavy metal exposure***. Infant exposure to lead (Pb) was reported as another environmental factor contributing to AD through DNA methylation regulation. Developmental exposure of rodents to the heavy metal lead has been shown to increase APP and Aβ in aging brain (Basha et al., 2005). Study in aged monkeys showed that the group that was fed with Pb in their early life has a lower DNMT activity. The downregulation of DNMT activity thus results in the hypomethylation of several genes involved in Aβ formation such as APP and BACE, and causes the upregulation of APP, BACE, and Sp1 in turn, which finally results in Aβ formation and AD (Wu et al., 2008). These finding are confirmed by a genome-wide study on mice (Bihaqi et al., 2011) and an *in vitro* study (Bihaqi and Zawia, 2012). A latent early-life associated regulation (LEARn) model, which claims that environmental agents perturb gene regulation at early stage but do not have pathological results until later in life, was proposed to explain these phenomena (Lahiri et al., 2008; Lahiri and Maloney, 2012).

#### Parkinson's disease, huntington's disease, and amyotrophic lateral sclerosis

Compared to AD, the studies are relatively insufficient about the causal relationship between DNA methylation and other three neurodegenerative diseases as well as the causes of DNA methylation in these diseases. Despite paucity of evidences, a few studies showed that environmental factors such as exposure to toxicity like paraquat and previous physical and mental experiences like a history of being abused in childhood, are related to DNA methylation in these diseases. However, data we can search so far are so rough and random findings that no reliable conclusion but simply assumptions can be made from them. Therefore, we can see a promising field of interest waiting for further solid research findings to confirm the hypothesis that similar to AD, there is also a causal relationship between DNA methylation and the other three neurodegenerative diseases (PD, HD, and ALS).

### Perspectives

Expanding rapidly as it is, the field of DNA methylation and neurodegenerative diseases is facing three main challenges for further progress.

First of all, DNA methylation is an emerging field with many unclear issues. For instance, one may expect that the hypermethylation will result in the repression of a gene, however, some opposite findings have been reported, such as the coexistence of overexpression and DNA methylation of the *p16INK4a* gene (Kim and Sharpless, 2006). Without an accurate comprehension of the relationship between DNA methylation and the regulation of gene expression, much experimental evidence linking DNA methylation and neurodegenerative diseases may have been misinterpreted or missed. In addition, 5hmC, which used to be regarded as only an intermediate in DNA demethylation, was recently found to increase with age in the absence of 5mC changes and thus may serve as an epigenetic factor of AD (Chen et al., 2012). With the majority of current studies concentrated on normal CpG methylation, we cannot ignore that non-CpG methylation and 5hmC also play an important role in DNA methylation. Since it has been suggested that non-CpG methylation and 5hmC are dominant in mammalian brain development (Lister et al., 2013), and since 5hmC is implicated in aging and AD (van den Hove et al., 2012), further studies should include these specific cases in order to give us a better understanding of how the DNA methylation is exactly related to the pathologies of neurodegenerative diseases.

Another challenge is that DNA methylation patterns vary in different cells as well as different brain regions. Study in human frontal cortex showed that neurons and glial cells do not share a same DNA methylation profile, nor do different neurons (Iwamoto et al., 2011). Since each type of cells in nervous system plays a distinct role, research ignoring variation among cells may not be able to provide sufficient evidence for this topic. Moreover, different regions of the brain, which have various importance in neurodegenerative diseases, were shown to express genes differently (Twine et al., 2011). Therefore, conclusions cannot be reached from studies merely focusing on one particular region of the brain.

The third challenge is the limitation in research methods. Most evidence of DNA methylation modification in neurodegenerative diseases was obtained from postmortem studies. Although this method was shown to preserve DNA methylation patterns successfully (Barrachina and Ferrer, 2009), it can only demonstrate the circumstance of one particular moment. Accordingly, the correlation between DNA methylation and neurodegenerative diseases can be revealed, but the time sequence of them is unable to be tracked. Such a limitation leads to the difficulty in determining the causal relationship of these events.

Hence, in the future, integrated and dynamic studies may significantly facilitate the development of this promising field. Comprehensive studies considering the difference among cell types and brain regions will certainly improve our understanding of this field. Furthermore, since DNA methylation is not an isolated process, studies must also consider interactions between DNA methylation and other epigenetic modifications such as histone acetylation. Although tracking the methylation status in a living animal cannot be achieved so far, it is possible to reach a dynamic perception via comparing postmortem results from samples with different ages, and such a strategy was used in some remarkable researches recently (Hon et al., 2013; Lister et al., 2013). Combined with the age-specific detection of neurodegenerative diseases, this type of studies will have great significance in uncovering the pathogenesis of neurodegenerative diseases, especially in the analysis of causal relationship between DNA methylation and such diseases.

## Conclusion

In sum, the correlation between DNA methylation and neurodegenerative diseases has been pointed by numerous studies. These findings further indicated that DNA methylation alteration is one of the causes for neurodegenerative diseases (Table 1). As we discussed, environmental factors, as well as other possible factors like aging, are responsible for these alterations and may guide us to a new path of treating neurodegenerative diseases. Finally, this area is still filled with unsolved problems and waiting for further investigations to reveal these secrets, in order to give us a more comprehensive knowledge of how DNA methylation is involved in neurodegenerative diseases.

**Table 1:** **Altered DNA methylation profiles observed in neurodegenerative diseases**.

**Disease**	**Category of genes**	**Specific genetic loci**	**DNA methylation regulation**	**Possible effect**
AD	Aβ-related genes	*APP*	Hypomethylation or none	Over expression of Aβ
		*PSEN1*	Hypomethylation	
		*BACE1*	Hypomethylation	
		*APBA2*	Hypermethylation	
	Aβ-degradation-related genes	*NEP*	Hypermethylation	Aβ accumulation
		*SORL1*	Hypermethylation	
	Tau-related genes	*GSK3β*	Hypomethylation	Disorder of tau
	Genes involved in metabolic pathways	*OTC*	Hypomethylation	Metabolic dysfunction
	rDNA	rDNA	Hypermethylation	Ribosomal deficits
	Others	Circadian gene (*PER1 and CRY1*)	Hypermethylation	Behavioral and psychological symptoms
		*LINE-1*	Hypermethylation	Unknown
		*NGB*	Unknown	AD severity regulation
		Telomere	Hypomethylation	Cell entering into a senescent state
		*COX-2*	Hypomethylation	Brain AA cascade enzymes upregulation
		*BDNF*	Hypermethylation	Loss of neurotrophic factors
		*CREB*	Hypermethylation	Exacerbation of BDNF reduction
		*SYP*	Hypermethylation	Loss of synaptic proteins
		*NF-kB*	Hypomethylation	Neuroinflammation
		*S100A2*	Hypomethylation	Protein accumulation
		*SORBS3*	Hypermethylation	Cell adhesion dysfunction
PD	*SNCA*	*SNCA* (intron1 and promoter)	Hypermethylation	Decreased expression of *SNCA*
			Hypomethylation	Overexpression of *SNCA*
	Inflammatory cytokines	*TNF-α*	Hypomethylation	Increased risk of apoptosis in dopaminergic neurons
	Clock genes	*CRY1 NPAS2*	Devoid of methylation	Disorder of circadian rhythms
	Telomere	Subtelomeric region	Constant methylation	Telomere shortening
	Other genes	*PARK16/1q32 GPNMB STX1B*	Methylation alteration	PD risk
		Cytochrome P45 2E1	Hypomethylation	Increased PD susceptibility
HD	*HTT* gene	Promoter region of *HTT* gene	Extensive methylation alteration	Neurogenesis and cognition
	Oligodeoxyribonucleotides	Cytidine	Hypermethylation	Expansion of triplet repeat sequences
	Genome	Genome-wide	Hypomethylation	Instability of CTG/CAG trinucleotide repeats
	*CTCF*	*CTCF* binding sites	CpG methylation	
	*BDNF*	Promoter region of *BDNF*	Gender-specific methylation	Differential regulation of *BDNF* gene expression
ALS	*SOD1 VEGF*	Promoter regions of *SOD1* and *VEGF*	Hypomethylation	No transcriptional silencing
	*EAAT2*	Promoter regions of *EAAT2*	Hypermethylation	Functional loss of *EAAT2* transporters
	*GLT1*	Promoter region of *GLT1*	Hypermethylation	
	*ATXN2*	Promoter region of *ATXN2*	Hypermethylation	Pathogenic CAG expansions

### Conflict of interest statement

The authors declare that the research was conducted in the absence of any commercial or financial relationships that could be construed as a potential conflict of interest.
